# Octopus engineering, intentional and inadvertent

**DOI:** 10.1080/19420889.2017.1395994

**Published:** 2017-12-14

**Authors:** David Scheel, P. Godfrey-Smith, S. Linquist, S. Chancellor, M. Hing, M. Lawrence

**Affiliations:** a Marine & Environmental Sciences, Alaska Pacific University, Anchorage, AK, USA; b Unit for History and Philosophy of Science, University of Sydney, Sydney, NSW, Australia, and CUNY Graduate Center, New York, NY, USA; c Philosophy, University of Guelph, Guelph, Canada; d Department of Biological Sciences, University of Illinois at Chicago, Chicago, IL, USA; e Centre for Sustainable Ecosystems Solutions, School of Biological Sciences, University of Wollongong, Wollongong, Australia; f

**Keywords:** *Octopus tetricus*, social, shelter, ecosystem engineering

## Abstract

We previously published a description of discovery of a site where octopuses live in an unusually dense collection of individual dens near one another in a bed of scallop shells amid a rock outcrop. We believe the shell bed is an extended midden, accumulated over time by individual octopuses returning to their dens with food. Here we consider what aspects of material collection, den maintenance, and aggregation are intentional for the octopuses, versus inadvertent consequences of individual decisions. Collection of prey items, transport of prey to the den, den excavation, and collection and use of non-prey materials at the den appear to be intentional behaviors. The occurrence of many dens in close aggregation appears to be an inadvertent outcome of the availability of food and the risk of predation in the habitat. Popular media reports have described this site as an ‘city’ designed by octopuses, but that is not an accurate description of the site.

We recently published a note describing discovery of an underwater site inhabited by unusually large numbers of gloomy octopuses (up to 15, on visits so far) in close proximity.^1^ This is the second site of this kind discovered in the same area.^2^ At both sites, octopuses live in dens excavated in a bed of discarded scallop shells. We hypothesize that a positive feedback process has operated at both sites, perhaps more markedly at the first. This environment contains ample food for octopuses, but many predators and a paucity of good den sites. If one or a few good dens can be established, and these are used over years, scallop shells left at the site by foraging octopuses can accumulate and provide a better den-building material than the local soft sediment. Other octopuses can eventually build dens using these shells, and those octopuses bring in still more scallops. Given the persistence of this behavior at one small location, the result is a site at which a dozen or more octopuses can build dens, and end up living in unusually concentrated circumstances.

In a series of news reports written about our paper, a tendency has grown to exaggerate what we reported. The site was described as “an underwater city” of octopuses (*Science, The Guardian*), as “an underwater city engineered by octopuses,” (*Quartz*), and as a demonstration that “octopuses are building underwater ‘cities’” (*Discover*). Another report has claimed, with no basis in our papers, that octopuses at these sites are “making art” out of shells (*BigThink*). Some exaggerations in these reports probably derive in part from the nicknames we have given to these sites (*Octopolis* for the first, *Octlantis* for the second), though these names are not used in our scientific reports. Our earlier work also described the octopuses as “ecosystem engineers.” This, in contrast, is not a nickname or metaphor, but use of an ecological concept that applies broadly to cases where organisms construct and transform their environments, especially in ways that modulate the availability of resources for the engineer species and/or others.^3^ Animals with complex behavior can be ecosystem engineers, but so also can plants and microbes.^4^ Ecosystem engineering does not require that the engineers *intend* to change their environments. The octopuses at these sites, by bringing in shells, have engaged in this kind of engineering, and their behavior has altered the living circumstances not only of the octopuses themselves but of many other local species.^5^

We want to take the opportunity not only to correct some misconceptions about our findings, but also to explore a question: what is the likely mix of intentional and non-intentional effects at these sites? How similar are these phenomena to those seen when animals build a structure (e.g. a bee hive or other communal nest) that they collectively inhabit?

The two sites we study contain a large number of dens, which come and go as they are maintained or fall into disuse. The dens are primarily holes in the shell bed, sometimes with barricaded collections of other objects around the lip (Fig. 1). These objects can be other kinds of biotic material (such as sponge fragments or larger shells), or beer bottles, research cameras, fishing gear, and other bits of human refuse. Some of the dens observed were narrow vertical shafts over 40 cm deep, lined with shells (unpublished measurement by PGS). A vertical shaft of 40 cm in the soft sediment alone is unlikely to be stable e.g.^6^ The shells make high quality dens possible (Fig. 1b).

**Figure 1.**
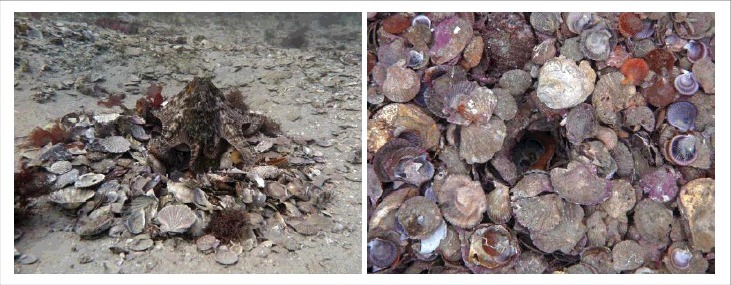
Two dens at the Octopolis site. (a) A den surrounded by shells at the periphery of the site. (b) A deep shaft-like den, lined with shells and occupied by an octopus.

What guides the actions of individual octopuses? When an octopus excavates a den, we might say that it intends to create a shelter. The notions of “intention” and “intentional behavior” are controversial and elusive ones. In the paradigmatic cases, an intention is an internal cognitive state that guides action by explicitly specifying an objective or goal. Octopuses may exhibit this kind of cognitive goal when they navigate around obstacles to reach a target that is temporarily blocked from view by the obstacle,^7^ or when returning to a previously used den.^8,9^ Many animal behaviors that appear intentional can, however, arise from mechanisms in which goals are not cognitively represented. Similar effects can be due to behavioral programs shaped by natural selection in a way that gives rise to an “as if” intentionality. A behavior that reliably achieves an adaptive outcome can often appear intentional. Examples include the building of nests by colonies of ants, and also the tracking of the sun's movements by some plants during the day.

There is unlikely to be a sharp divide between these two categories – between genuine and merely “as if” intentions.^10^ Instead, the “edges” of intention are gradual. There are many relevant gradations in complexity, ranging from inflexible but adaptive responses that resemble reflexes, through adaptive behaviors modified only by perceptual tracking of the details of current conditions, through behaviors shaped by instrumental learning, all the way to behaviors guided by the explicit formulation of a goal.^11^ A distinction between intentional and non-intentional might be used to mark out various distinctions across this range. In the present context, the contrast that is especially relevant is that between intended and inadvertent outcomes of behaviors. We will say that a behavior is *intentional* if it is the product of internal states that guide action towards an adaptive or internally represented outcome in a way that is sensitive to environmental and/or proprioceptive feedback, such that the behavior is modified in response to requirements imposed by local contingencies, and changes or ceases when the “intended” outcome is achieved. This contrasts with behaviors that achieve an effect that the animal does not represent, and where feedback from that outcome does not modify what the animal does. “Fixed action patterns,” while controversial (e.g.^12^), may be insensitive to environmental feedback and hence not intentional in our sense; the classic example is egg rolling behavior by graylag geese.^13^ If the egg is removed from under the bird's bill before the behavior has returned the egg to the nest, the goose nonetheless completes the entire behavior.

Clearing a hole for shelter may be a behavior in which this goal is not explicitly represented by the octopus. However, the octopuses do sometimes fastidiously maintain and extend their dens by expelling algae, silt, and shells that may be collected from deep in the den and propelled some distance away with the aid of a jet from the animal's siphon, or carried some distance from the den before being discharged. We do not see octopuses going through stereotyped cleaning behaviors with no debris in their grasp. When shells and other materials being arranged fall back into the den, octopuses may repeat and adjust behaviors directed on the objects (though several explanations may be available for such actions). Den building and maintenance by these animals is quite a complex behavior and nothing like a simple reflex (by contrast, see e.g.^14^).

Each octopus that builds a den does so individually; there is no teamwork or collective action as far as we know. We also have not found two octopuses co-occupying a den at these sites (though dens may be in very close proximity – individuals in separate dens may be as little as 20 cm apart, measured from the center of each animal).

What is it about the collecting of the shells that makes dens of this kind possible? Octopuses at these sites forage for scallops and bring them back to their current den to eat. It is important to note that this behavior is not unique to this population. Many other octopus species discard prey remains in a pile outside their dens. Remains have long been used by researchers to locate octopus dens and to gauge their individual prey choices (e.g.^15–18^). At these sites, the shells are left lying around the den of the octopus who collected that meal, or sometimes thrown some distance from a den. The composition of material around the den may be actively maintained^19^ (see Fig. 2 for images of an octopus collecting, installing and using a piece of sponge both as a barricade and “hatch” over a den shaft.)

**Figure 2.**
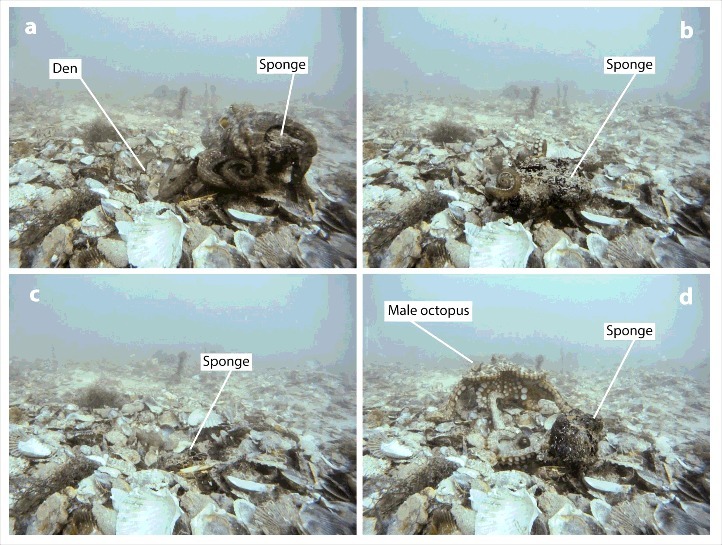
A sequence of video screenshots from the Octopolis site. (a) An octopus brings a piece of sponge back from a location off-camera to the right. The octopus left the den to the right, was absent for approximately 30 seconds, and returned carrying the sponge. (b) The octopus holds the piece of sponge at the edge of the den. The sponge is placed directly between the octopus and the unmanned GoPro camera recording this sequence. (c) Later, the octopus in its den pulls the sponge over the top of the den opening, below the level of the shell bed. (d) The octopus is mated by a male who approaches the den. The hectocotylized third right arm of the male is beneath a single arm of the female, which is extended to the male.

As yet, we have no reason to believe that the bringing of scallop prey back to a den and discarding of the shells is *intended* to alter the habitat. We think this effect is probably an inadvertent consequence of the fact that eating at home, for an octopus, is safer than eating where the scallop was found. Eating in the open risks attention from predators, and excessive time spent away from the site might lead to one's den being occupied by another octopus. Foraging, which requires an active search by the octopus, and the bringing of scallops to the den are intentional behaviors in the broad sense we introduced above. So are at least some behaviors associated with building and maintaining dens. A burrowing movement in response to a perceived threat might be non-intentional or barely intentional, but the arrangement of objects around a den entrance and the attentive removal of debris are probably intentional behaviors. There need be no representation in the octopus of some important relations between their actions here – no insight into the fact that bringing scallops home to eat will improve the den building possibilities for many octopuses at the site. The accumulation of shells is an inadvertent long-term product of many individual behaviors that may well be intentional, but serve only short-term individual goals.

An interesting question is whether the fact that a scallop shell can function as building material ever plays any role in an individual octopus bringing that scallop back to the site. As Fig. 2 shows, octopuses do actively bring inedible material back to their dens at this site, apparently to serve as barricades. Finn, Tregenza and Norman^20^ also showed that octopuses will carry around quite ungainly inedible objects – coconut shells – for use as shelter when the need arises. The octopuses at Octopolis harvest the larger scallops among those available.^5^ While large shells could be better materials for den construction, larger scallops also provide a larger meal, and there have been large numbers of shells available at the site for quite a few years now. It is possible, therefore, that live scallops at these sites are viewed only as food, and the availability of the discarded shells for den building is a fortuitous byproduct.

Finally, the octopuses at these sites are not building cities. An aggregation of individual dwellings, even where each is intentionally constructed, is not a city. A city is a center not just of population but of commerce, culture and design. Cities are cooperatively constructed and maintained communities. We are not making this claim for *Octopus tetricus*.
